# Peer-Mentored Social Media Groups for Youth Vaping Cessation: Single-Arm Pilot Trial

**DOI:** 10.2196/46283

**Published:** 2026-05-25

**Authors:** Joanne Chen Lyu, Justin S White, Kaira Shlipak, Aliyyat Afolabi, Pamela M Ling

**Affiliations:** 1 TSET Health Promotion and Research Center, University of Oklahoma Health Sciences, 655 Research Parkway Suite 400, Oklahoma City, OK, 73104, United States, 1 405 271 6872; 2 Department of Family and Preventive Medicine, College of Medicine, University of Oklahoma Health Sciences, Oklahoma City, OK, United States; 3 Center for Tobacco Control Research and Education, University of California, San Francisco, San Francisco, CA, United States; 4 Department of Health Law, Policy and Management, Boston University School of Public Health, Boston, MA, United States; 5 Philip R Lee Institute for Health Policy Studies, University of California, San Francisco, San Francisco, CA, United States

**Keywords:** peer mentoring, vaping cessation intervention, social media, adolescents, young adults

## Abstract

**Background:**

Using social media to deliver e-cigarette cessation interventions to young people is a promising approach, but low participant engagement and retention may undermine intervention efficacy. Peer mentoring holds great potential to address these issues.

**Objective:**

This study aimed to understand the feasibility and acceptability of integrating peer mentoring into a social media–based intervention to help young people quit e-cigarette use.

**Methods:**

We conducted a single-arm pilot trial with 24 e-cigarette users (aged 16‐21 years). Participants were assigned to 1 of 2 Instagram groups, where they received an existing vaping cessation intervention, Quit the Hit (QTH), in which a trained counselor posted 1 to 3 evidence-based vaping cessation messages on weekdays over 5 weeks. As an adjunct to QTH, each group included 2 peer mentors of similar age as participants who had successfully quit vaping. The mentors provided social support and shared their quitting experiences. Participants completed a baseline survey and a follow-up survey at the end of the intervention (week 5) and were invited to participate in postintervention focus groups.

**Results:**

Participants’ mean age was 18.6 (SD 1.2) years, and 58.3% (14/24) were male. Most (20/24, 83.3%) identified as straight/heterosexual, 29.2% (7/24) identified as non-Hispanic White, and 45.8% (11/24) were high school students. On average, participants vaped e-cigarettes 5.1 (SD 1.7) days per week, and 33.3% (8/24) reported daily vaping. All participants (24/24, 100%) had made at least one quit attempt in the past year. Nearly all participants (23/24, 95.8%) engaged in the group by either posting or liking others’ comments at least once. Mean program engagement was 28.8 (SD 27.3) posts or likes over the 5 weeks. The study retention rate was 91.7% (22/24) at week 5. Using an intent-to-treat approach, 66.7% (16/24) of participants were abstinent (7-day point prevalence abstinence) at week 5. On a 5-point Likert scale, participants rated peer mentoring as highly useful (mean 4.6, SD 0.3) and reported high levels of satisfaction (mean 4.7, SD 0.3). All participants who completed the follow-up survey said they would recommend the program to others. Focus group findings supported these results, highlighting positive feedback on both the quality and usefulness of peer mentoring and the peer-mentored version of the QTH program. Suggestions for future improvements were also discussed.

**Conclusions:**

Integrating peer mentoring into social media–based e-cigarette cessation interventions for young people is feasible and acceptable. Participants engaged in the intervention and demonstrated strong retention. They rated both the peer mentoring and the overall program highly. Self-reported abstinence at 5 weeks was high. Larger trials are warranted to evaluate the efficacy of the peer-mentored intervention.

## Introduction

As cigarette smoking has declined after decades of tobacco control efforts, e-cigarettes have become the most commonly used tobacco product among adolescents and young adults [1,2]. Among US adults, young adults reported the highest rate of e-cigarette use, with 11% to 18% reporting vaping in the past 30 days [3-5]. According to the 2024 National Youth Tobacco Survey, 5.9% of middle and high school students (1.6 million) reported current e-cigarette use [6]. While the long-term effects of e-cigarette use are not yet fully understood, evolving evidence indicates an increased risk for respiratory and cardiovascular diseases [7-9]. In addition, early exposure to nicotine may increase the risk of addiction [10,11] and the likelihood of initiating cigarette smoking later in life [12-14]. A meta-analysis published in 2018 found that, after adjusting for established risk factors for cigarette smoking, youth e-cigarette users had nearly 4 times higher odds of subsequent cigarette smoking [15]. More recent meta-analyses of longitudinal studies have further confirmed the association between e-cigarette use and subsequent smoking among nonsmoking adolescents and young adults [14]. Quitting tobacco before age 30 reduces most tobacco-related health risks [16]; therefore, it is crucial to provide effective vaping cessation support for adolescents and young adults [17].

The growth of social media use among young people [18] makes it a potentially ideal channel for delivering vaping cessation interventions to adolescents and young adults [19,20]. Previous studies on social media–based smoking cessation interventions have shown feasibility, acceptability, and early efficacy [19]. However, despite these promising findings, social media interventions have reported large reductions in engagement over time and high dropout rates [19,20]. For instance, the Tobacco Status Project (TSP), a smoking cessation intervention for young adults delivered via Facebook groups, found that the number of participant comments decreased over time in each group despite variation between groups [21]. User engagement with cessation interventions has been found to be associated with retention and abstinence outcomes [22,23], highlighting the need to improve participant engagement to enhance the effectiveness of social media interventions.

The scientific literature suggests that peer mentoring may be a promising strategy to engage participants and address the issue of decreasing engagement and low retention in social media–based interventions. The model of supportive accountability [24], grounded in organizational psychology, motivation theory, and computer-mediated communication research, posits that human support from a trustworthy, benevolent, and competent source helps digital intervention participants stay engaged and adherent to interventions by offering social support and accountability. It also suggests that peer mentors can be especially effective because individuals often feel more at ease with those who are similar to themselves [24]. As a peer support strategy, peer mentoring is a process in which individuals with similar life experiences provide encouragement and assistance to foster health behavior change [25]. Previous studies with young people have shown that tobacco use is susceptible to peer influence [26-28]. Incorporating peer mentoring into vaping cessation programs may be effective because peers who have successfully quit are more relatable than health care professionals or adult facilitators [25,29]. Peers’ personal experiences may show that cessation is achievable, and their social support may be more engaging for participants [30]. Peer mentoring has been adopted by various health promotion programs [31-33], including smoking cessation programs [34,35], yet it has not been used to address e-cigarette use in the context of social media. In this study, we aimed to evaluate whether integrating peer mentoring into social media–based interventions to support e-cigarette cessation among young people was feasible and acceptable.

## Methods

We conducted a single-arm pilot trial that integrated peer mentoring into an existing Instagram-based vaping cessation program, Quit the Hit (QTH), to assess feasibility and acceptability quantitatively. Postintervention focus group discussions were conducted with participants to contextualize the quantitative findings.

### Intervention Design

QTH is a 5-week program that delivers evidence-based vaping cessation content in groups via direct messages on Instagram. QTH was developed by a University of California, San Francisco research team in partnership with outside agencies (HopeLab and Rescue) and based on the TSP discussed above, which showed efficacy in delivering a smoking cessation intervention to young adults in private Facebook groups [36]. QTH convenes Instagram direct-message groups of 10 to 15 participants facilitated by a professional tobacco cessation counselor; the counselor delivers up to 3 posts per weekday. Content includes evidence-based vaping cessation strategies, incorporating skills from cognitive behavioral therapy and the transtheoretical model that have demonstrated efficacy for long-term smoking cessation [37]. Additional details about QTH have been published previously [38].

Peer mentoring was provided by young adults aged 18 to 29 years who had used e-cigarettes, had previously participated in QTH support groups, and had successfully quit vaping for more than 3 months. This 3-month threshold was used because abstinence at 3 months is a commonly reported benchmark in tobacco cessation research for “successful” short-term sustained cessation [39], although it does not eliminate the possibility of relapse. Peer mentors received a 4-module mentor training (basic information about e-cigarettes and health risks, mentoring relationships and skills, emotional intelligence, and boundary setting and confidentiality) and passed a posttraining qualification assessment that included a knowledge test and scenario-based questions. The mentoring relationships and skills module was a live Zoom training with a trained counselor, while the other modules were provided as recorded trainings that mentors completed on their own time. The training modules were adapted from a previous peer-mentored smoking cessation intervention and informed by our formative research on mentors’ training needs and participants’ expectations [35,40] and consultations with experts in vaping cessation, peer mentoring, and youth-serving programs, with the goal of preparing mentors to effectively fulfill their mentoring roles.

**Figure 1.**
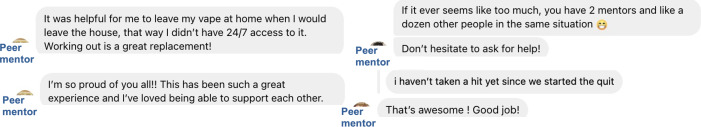
Screenshots of peer mentor posts in Instagram groups illustrating emotional support.

Peer mentors posted in the QTH groups to provide emotional support, often sharing their personal experience with quit attempts and offering encouragement. Figure 1 shows examples of peer mentor posts in the QTH groups. In each peer-mentored QTH group, there were 8 to 12 participants, 2 peer mentors, and a professional tobacco cessation counselor. Participants could direct-message their mentors to receive one-on-one peer mentoring if they were uncomfortable reaching out in the group setting. Peer mentors sent each participant a check-in message around the third week. Program features, such as the age range of peer mentors, group size, and mentor-mentee ratio, were informed by our formative study [40].

### Participants and Recruitment

Participants were recruited through social media advertising (Meta Ads, including Facebook and Instagram, and TikTok advertising), recruitment messages distributed by youth-serving community organizations, and outreach to participants of a previous randomized controlled trial (RCT) testing QTH [38] who did not quit vaping by the end of the trial. The recruitment message included a link and QR code to the study’s website, where eligibility was screened. Eligible participants were able to read English, aged 13 to 21 years, used social media at least 4 days per week, vaped at least once per week in the past 30 days, indicated that they were considering or interested in quitting within the next 6 months, and resided in California. Participants were asked to show photos of their ID and vape devices to verify their age and vaping status during study enrollment.

### Study Procedures

After giving consent and completing a baseline survey, participants were assigned to one of the 2 support groups based on timing of enrollment. The first 12 participants were assigned to group 1, and the next 12 were assigned to group 2. After participants joined the group, we reviewed the ground rules with participants, including expectations for respectful behavior and confidentiality (ie, what is said in the group stays in the group). The intervention lasted 5 weeks. At the end of the intervention, participants were asked to complete a follow-up survey and were invited to participate in a 90-minute focus group via Zoom. The focus group discussion asked participants to reflect on their experiences with peer mentoring and the overall intervention and to provide suggestions for future improvements. Focus group discussions were video recorded and professionally transcribed.

### Measures

#### Overview

Both baseline and follow-up surveys were administered online via Qualtrics (Qualtrics, Inc). Participants reported their sociodemographic characteristics, e-cigarette use, and quitting-related factors, such as quit attempts, desire to quit, and confidence in quitting. Measures of feasibility (including engagement and retention) and acceptability (including perceived usefulness of peer mentoring, satisfaction with peer mentoring, and evaluation of the program) were adapted for use in a vaping cessation context based on prior pilot trials for smoking cessation with and without peer mentors [35,38,41].

#### Feasibility

Feasibility was assessed by participant engagement in the Instagram group, as indicated by the number of likes and comments documented by study staff, which is consistent with previous social media interventions [41] and the retention rate. Retention was measured as the proportion of enrolled participants who completed the follow-up survey.

#### Perceived Usefulness of Peer Mentoring

Perceived usefulness was assessed in the follow-up survey with 9 items, measuring agreement on a 5-point Likert scale (1=disagree a lot to 5=agree a lot). Example items included the following: “The peer mentors helped me quit vaping,” “The peer mentors shared useful information on quitting,” and “The peer mentors increased my confidence that I can quit.”

We calculated an index of the overall perceived usefulness as the mean of responses across all 9 items.

#### Satisfaction With Peer Mentoring

Satisfaction with peer mentoring was assessed in the follow-up survey with 6 items, measuring agreement on a 5-point Likert scale (1=disagree a lot to 5=agree a lot). Example items included the following: “I liked the peer mentoring in the program,” “I felt comfortable with the messages I got from peer mentors,” and “The peer mentors sent messages at convenient times of day.”

Additionally, participants rated their satisfaction with the quantity of peer mentor posts on a 5-point scale by responding to the item, “Overall, the number of messages from peer mentors was …” (1=too few, 2=few, 3=about right, 4=many, and 5=too many).

#### Evaluation of the Program

The overall evaluation of the peer-mentored vaping cessation program was measured with 4 items, measuring agreement on a 5-point Likert scale (1=disagree a lot to 5=agree a lot). The 4 items were the following: “I would recommend this program to others,” “The program gave me something new to think about,” “I thought about what I read in the program” and “The program was easy to understand.”

### Data Analysis

We summarized descriptive statistics for participant characteristics, feasibility, and acceptability as indicated by perceived usefulness, satisfaction, and evaluation of the program, and other variables of interest. A series of comparisons about e-cigarette use were made between preintervention and postintervention data, but given the small sample size, no inferential statistics were used. All analyses were conducted using SPSS (version 26; IBM Corp).

We adopted a thematic analysis approach to analyze qualitative data from 2 focus groups (5 participants total), with the assistance of Dedoose software (SocioCultural Research Consultants). Using a data-driven inductive approach, one author (JCL) reviewed all transcripts from both groups and developed the initial coding guide. With this guide, 2 authors (JCL and KS) independently coded the transcripts and resolved any discrepancies through discussion. Analytic memos were written during the independent coding phase and later discussed to refine the coding guide. Given the focus of this study and limited number of focus group participants, thematic saturation was not the goal of the qualitative analysis; instead, we aimed to identify key points expressed by participants regarding their experiences in the program to contextualize the quantitative findings.

### Ethical Considerations

This study received approval from WIRB-Copernicus Group Institutional Review Board (protocol T31IR1910). The trial was registered at ClinicalTrials.gov (NCT07437196). Participants completed online informed consent, consistent with standard care for adolescents in smoking cessation treatment [42]. Participants received electronic gift cards for completing the baseline survey and follow-up survey. They received US $10 for completing the baseline survey and US $20 for completing the follow-up survey, with a US $5 bonus per survey for completion within 24 hours of receiving the survey link. Participants who completed the 90-minute postintervention focus group received US $50. Peer mentors received US $30 for completing the saliva test for biochemical verification of abstinence and US $50 for completing peer mentor training and passing the subsequent assessment. They also received US $250 at the end of the 5-week program for their effort.

## Results

### Participant Characteristics

Among the 24 participants, 15 were recruited via TikTok ads and 9 were recruited from a list of prior QTH RCT participants who met eligibility criteria, including 7 from the control group and 2 from the intervention group. Table 1 shows the details of the participant characteristics. Participants were predominantly male (n=14, 58.3%). While participants aged 13 to 21 years were eligible, the enrolled participants ranged in age from 16 to 21 years, with a mean age of 18.6 (SD 1.2) years. Most participants (n=20, 83.3%) identified as straight or heterosexual, 7 (29.2%) identified as White, and almost half (n=11, 45.8%) were high school students. All participants reported vaping in the past 7 days, with an average of 5.1 (SD 1.7) days per week, and 8 (33.3%) participants reported vaping daily. On vaping days, the average number of puffs was 31.6 (SD 62.5). A total of 8 (33.3%) participants reported using e-cigarettes within 30 minutes of waking. Overall, participants reported strong motivation to quit. All participants (n=24, 100%) had made at least one quit attempt (ie, stopped vaping nicotine for 1 day or longer) within the past 12 months, and on a 1- to 5-point scale (5=very much), the average desire to quit was 4.5 (SD 0.6), and the average confidence in quitting was 4.0 (SD 1.0).

**Table 1.** Participant characteristics (N=24).

Characteristics	Values
Sociodemographic characteristics	
Age (years), mean (SD)	18.6 (1.2)
Sex at birth, n (%)	
Female	10 (41.7)
Male	14 (58.3)
Sexual identity, n (%)	
Heterosexual	20 (83.3)
LGBTQ+^a^	4 (16.7)
Race and ethnicity, n (%)	
Hispanic	6 (25)
Non-Hispanic Asian	1 (4.2)
Non-Hispanic Black	1 (4.2)
Non-Hispanic White	7 (29.2)
Other or multiracial	9 (37.5)
Educational attainment, n (%)	
High school	11 (45.8)
Not enrolled	2 (8.3)
GED^b^ classes or community college	6 (25)
4-year college or university	5 (20.8)
e-Cigarette use characteristics	
Puffs per day, mean (SD)	31.6 (62.5)
Days per week vaped, mean (SD)	5.1 (1.7)
Daily vaping, n (%)	8 (33.3)
Time to first puff after waking, n (%)	
Within 5 min	3 (12.5)
6‐30 min	5 (20.8)
31‐60 min	12 (50)
After 60 min	4 (16.7)
Quit-related characteristics	
Past-year quit attempts, n (%)	24 (100)
Desire to quit, mean (SD)	4.5 (0.6)
Confidence in quitting, mean (SD)	4.0 (1.0)

a LGBTQ+: lesbian, gay, bisexual, transgender, queer or questioning, or others.

b GED: General Educational Development.

### Feasibility: Engagement and Retention

Almost all participants (23/24, 95.8%) engaged by posting or liking comments in the group at least once during the program. Combining posting and liking, participants engaged with the program a mean of 28.8 (SD 27.3) times and median 15.5 (IQR 9.0-44.8) times over the 5 weeks. There was no substantial difference between group 1 (median 18.0, IQR 9.3-61.3) and group 2 (median 13.0, IQR 9.0-44.3). Specifically, of the 24 participants, 23 (95.8%) posted at least once during the program. The mean number of posts per person was 18.6 (SD 18.5), and the median number of posts was 10.0 (IQR 8.0-26.3). Excluding the 1 participant who never posted, individual post counts ranged from 1‐67. A total of 21 (87.5%) participants ‘‘liked’’ at least one post in the group. The mean of “likes” per person was 10.3 (SD 11.1). The median number of “likes” was 5.5 (IQR 1.0-18.8). Among those who “liked” at least once, individual “like” counts ranged from 1‐38. At week 5, 22 (91.7%) participants remained in the group and completed the follow-up survey.

### Perceived Usefulness of Peer Mentoring

**Figure 2.**
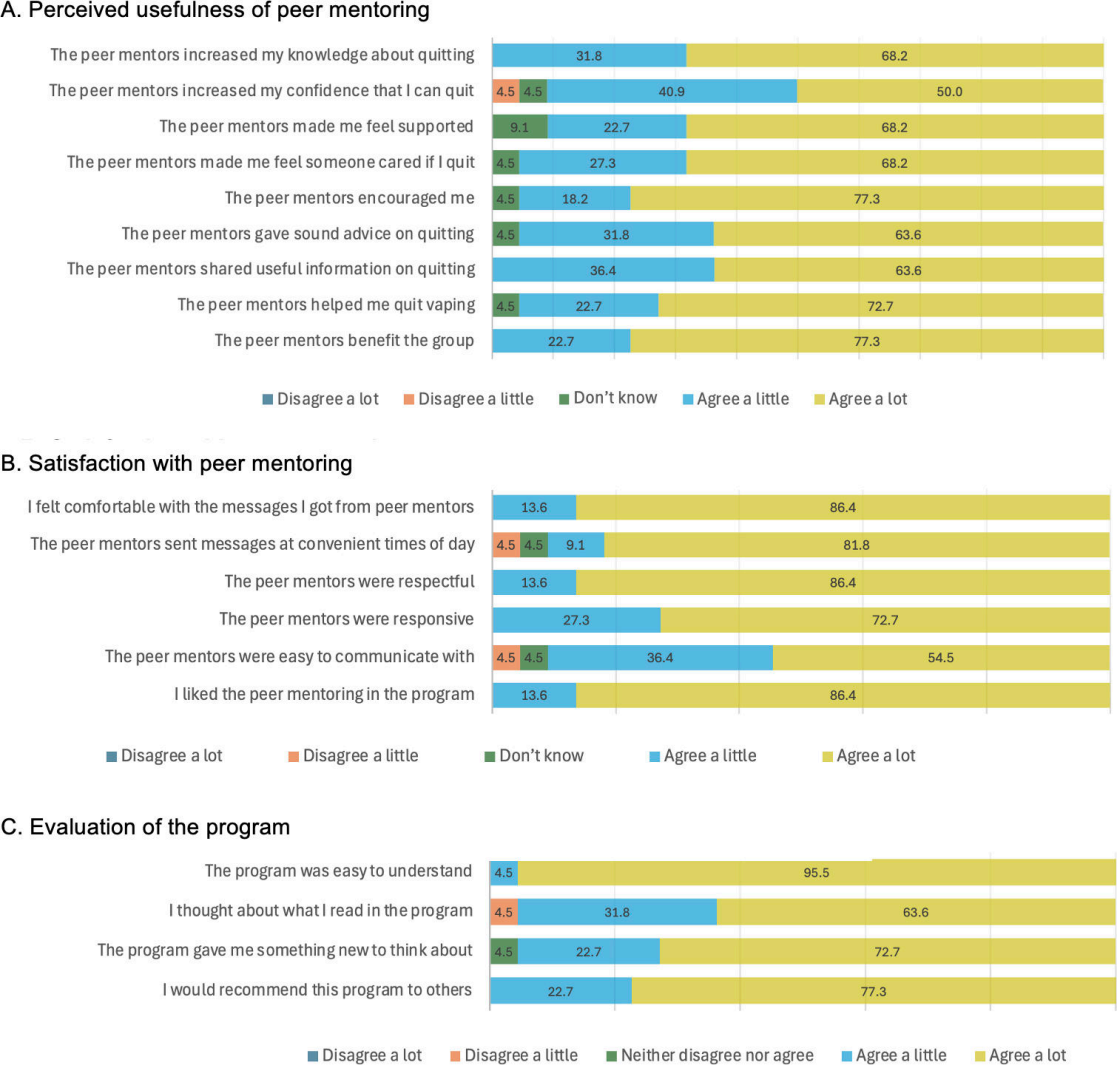
Proportion (%) of responses for acceptability measures. (A) Perceived usefulness of peer mentoring; and (B) satisfaction with peer mentoring; (C) evaluation of the program.

Peer mentoring provided in the group was perceived to be highly useful (Figure 2A). All participants agreed that their peer mentors increased their knowledge about quitting, provided useful information, and benefited their group. Nearly all participants (21/22, 95.5%) agreed that the mentors showed care, offered encouragement, gave good advice, and helped them quit vaping. A total of 90.9% (20/22) of participants reported that the mentors boosted their confidence in quitting and made them feel supported. Across the 9 items measuring perceived usefulness, the mean score was 4.6 (SD 0.3) out of 5.

### Satisfaction With Peer Mentoring

The participants expressed very high levels of satisfaction with the peer mentoring they received in the group (Figure 2B). All participants agreed that they liked the peer-mentoring component, felt comfortable with the messages they received, and found the mentors respectful and responsive. Most (20/22, 90.9%) participants also agreed that the mentors sent messages at convenient times and were easy to communicate with. Across the 6 items measuring satisfaction with peer mentoring, the mean score was 4.7 (SD 0.3) out of 5. In addition, 86.4% (19/22) of participants felt the number of messages from peer mentors was “about right,” 9.1% (2/22) thought there were “few,” and 4.5% (1/22) thought there were “many.” No participant selected the options of “too few” or “too many.”

### Evaluation of the Program

The participants evaluated the peer-mentored vaping cessation program highly (Figure 2C). All participants agreed with the statements “I would recommend this program to others,” and “The program was easy to understand.” Nearly all participants (21/22, 95.5%) agreed that “The program gave me something new to think about,” and “I thought about what I read in the program.”

### Vaping Abstinence Outcomes

Using an intent-to-treat approach, 66.7% (16/24) of participants reported being abstinent at the end of the program based on 7-day point prevalence abstinence. Of the participants who completed the follow-up survey, 72.7% (16/22) reported being abstinent at 5 weeks. Among participants who had not quit, the average number of vaping days per week was 1.8 (SD 1.2) days, lower than the average of 5.1 (SD 1.7) days per week at baseline, and no participant reported daily vaping at follow-up. Similarly, the average number of puffs per vaping day decreased to 5.7 (SD 4.7) puffs, compared with 31.6 (SD 62.5) puffs at baseline.

### Focus Group Findings

We conducted 2 postintervention focus groups with 5 participants from the 2 groups (1 female and 4 male). Three participants reported abstinence and 2 did not, and 3 had participated in a prior QTH group without peer mentors. The following themes were identified: appreciation of having peer mentors, communication with mentors, feedback on program setup, privacy and boundaries, advice for improvement, and willingness to be a future mentor.

All 5 participants expressed high satisfaction with the peer mentoring they received, valuing both mentors’ approachable communication style, characterized by respect and responsiveness, and their steady support. One participant noted the following (theme: appreciation of having peer mentors):

*I feel like they’re the most helpful with just general support. I have the group as community support overall, but the peer mentors serve as kind of an anchor... I feel like the peer mentors were really stable source of support in the group and I feel like that was a benefit.*

Some participants who had previously participated in a QTH group without mentors recalled that a large number of participant posts off the topic of vaping cessation discouraged participation, and this was improved with peer mentors:

*I had muted it around week two... every hour that I would get a notification of 99 plus messages... the actual topics of conversation were not beneficial to quitting and we’re completely off of what should be discussed*.

By comparison, these participants felt that peer mentors’ presence in the group discussions kept the group focused and on track (theme: appreciation of having peer mentors). They were also satisfied with the number of times their mentor messaged them and the wait time before receiving a response (theme: communication with mentors). The group size and ratio of mentors to mentees were considered acceptable, as noted by one participant (theme: feedback on program setup):

*Both of my mentors were pretty active, so I always felt like they were around. So, I thought two was the perfect number.*

Participants appreciated being offered the options of peer mentoring both one-on-one and in the group setting (theme: feedback on program setup). They mentioned that the peer mentors fostered a welcoming environment in the group, which also facilitated comfort with one-on-one mentoring. Among the 5 focus group participants, 3 direct-messaged their mentors (themes: feedback on program setup and communication with mentors):

*I feel like the way he interacted in the group chat first also made me feel comfortable to reach out. He seemed very open*.

*It’s like you get to know them and the DMs, it’s a lot more personal, ... I like that we have both options instead of one.*

Although all participants agreed that one-on-one support was a valuable supplement to group discussions, they had mixed views on peer mentors proactively direct-messaging them. Most participants appreciated it and felt it increased their sense of connection to the group, with one participant noting the following:

*I thought that this program and the group chat was very helpful, especially the mentors, especially when they DM me personally, so I never felt that way of leaving the program, and they really helped us quit*.

However, one participant felt pressured by the DM and said they might have considered leaving the group if they received more (theme: privacy and boundaries).

Participants in the focus group also affirmed core components of the QTH program and believed some helped with the mentor-mentee relationship (theme: feedback on program setup). For instance, the counselor posted Monday roll calls as brief check-ins in the group chat that invited members to respond to a fun question or prompt. Participants felt that these posts helped maintain engagement, and they described the group-based design as both supportive and motivating. As one participant explained:

*I guess the motivation was just other people—seeing other people also trying to achieve the same goal as me. It just pushes you forward instead of relying on your own goals, so you get inspiration from other people*.

While participants wanted the program to focus on vaping cessation, they also appreciated that not all questions were strictly about vaping. For example, a question from QTH asked, “If you were a food what food would you be?” and a participant answered, “I’d be a grape because I want to be with my friends all the time.” They felt that having nonvaping-related interaction questions once a week was just the right amount and helped keep them engaged and connected with both their peers and the mentors.

When asked for suggestions to improve the program, participants recommended incorporating more personal interactions, such as video messages, video meetings, and designated office hours for real-time support (theme: advice for improvement). Several noted that a mentor had used a video self-introduction, which was very well received. As one participant shared:

*It seems like they sent you a personal message even though it’s sent to everyone. I feel like that [sending a video message] would help you get to know them just a little bit better*.

They also suggested that the QTH counselor share more resources during the program, particularly on Fridays, so they could review the materials over the weekend when there were no messages from the counselor in the group. Additionally, a couple of participants expressed a desire for a longer intervention than 5 weeks to further support abstinence. Participants did not indicate any further concerns with the peer-mentored QTH program, participant safety, or privacy. Two participants also expressed interest in serving as peer mentors in future cohorts (theme: willingness to be a future mentor).

## Discussion

### Principal Findings

Our study found that integrating peer mentors who had successfully quit vaping into QTH, an Instagram-based vaping cessation program, was both feasible and acceptable. While the study was not designed to formally assess efficacy, using an intent-to-treat analysis, 66.7% (16/24) of participants reported 7-day point prevalence abstinence. Postintervention focus groups provided qualitative data confirming that participants highly valued the peer mentoring in the program, found the peer mentors helpful in their quit efforts, and endorsed a peer-mentored approach. Together, these findings underscore the promise of peer-mentored, social media–based vaping cessation interventions and the need for future trials to assess their efficacy.

Overall, engagement in the peer-mentored QTH intervention was encouraging. QTH was adapted from the Facebook-based TSP smoking cessation intervention for young adults that showed efficacy. We used the same engagement metrics (ie, number of comments and likes) as in the TSP pilot lasting 90 days. In the TSP pilot, 61% (48/79) of participants commented on at least one post and 51% (40/79) liked at least one post, compared with 95.8% (23/24) and 87.5% (21/24), respectively, in our peer-mentored intervention. The median number of comments was higher in our intervention than in TSP (15.5 vs 12), and the median number of likes was also higher (5.5 in our intervention vs 4 in TSP) [41]. TSP did not report mean overall program engagement, limiting direct comparison on this metric. However, mean program engagement in the peer-mentored intervention was 28.8 (SD 27.3) posts or likes, which was lower than the mean of 47.5 posts or likes in the QTH RCT [43]. This may partly be explained by the large number of nonvaping-related engagement, as noted by our postintervention focus group participants who had been recruited from the QTH RCT and did not successfully quit. It should also be noted that participants reported sending DMs to their mentors during the peer-mentored program; however, one-on-one peer mentoring via DMs was not captured as an engagement metric because the research team could not access these messages outside the group. Therefore, we may have understated participant engagement in the peer-mentored QTH program.

Regarding retention at the end of intervention, TSP reported 76% (60/79) retention [41], the QTH group in an RCT without peer mentoring reported 48.4% (124/256) retention [44], and our QTH group with peer mentoring achieved 91.7% (22/24) retention. However, the program durations differed (90 days in TSP vs 5 weeks in QTH), and recruitment strategies also varied across studies; therefore, the presence of peer mentoring may not fully explain the differences in study retention. The purpose of incorporating peer mentoring as a source of social support was to enhance participant engagement, which is a key driver of retention. Although we could not statistically establish the relationships among these factors, both the observed engagement metrics and qualitative feedback from postintervention focus groups suggest that peer mentoring is a promising and well-received strategy for increasing topic-focused engagement in social media–based interventions. Further research is recommended to clarify these relationships.

We also found high levels of satisfaction with and perceived usefulness of peer mentoring among the participants. Participants expressed high levels of satisfaction, as indicated by high mean values. All participants who completed the follow-up survey agreed that their peer mentors “increased [their] knowledge about quitting,” “shared useful information on quitting,” and “benefited the group.” Focus group discussions echoed these findings, revealing that peer mentors benefited the group by keeping the conversation focused on quitting, compared with programs without peer mentoring. Participants also expressed strong satisfaction with the peer mentoring they received in the group, highlighting easy and comfortable communication, a respectful and responsive style, messages sent at convenient times and in appropriate amounts, and the effective combination of one-on-one and group mentoring. All participants agreed with the statement, “I like the peer mentoring in the program.” Participants’ positive responses to the current peer-mentoring approach reflect the success of incorporating our formative findings into program development and underscore the potential of this approach for future interventions [40]. Beyond the very positive perception of peer mentoring itself, participants also highly rated the peer-mentored version of the QTH program, with all indicating that they “would recommend this program to others.”

One consideration for implementing peer mentoring is the additional resources required to train, support, and compensate peer mentors, which increase program cost and complexity. However, this model remains more efficient than in-person peer-mentoring programs, which require more substantial resources [34]. Our participants’ strong evaluations of both the peer mentoring and the peer-mentored intervention as a whole highlight the potential of a geographically unrestricted, low-cost peer support model. Moreover, participants expressed interest in serving as peer mentors themselves, suggesting the potential to develop a large, sustainable mentor pool to support subsequent cohorts of individuals seeking to quit.

Though this pilot study focused on feasibility and acceptability, 72.7% (16/22) of participants who completed the follow-up survey reported abstaining at the end of the intervention. Using an intention-to-treat approach, the reported 7-day point prevalence abstinence rate was 66.7% (16/24), which is higher than the self-reported abstinence rate at the end of intervention in the QTH group without peer mentoring (67/256, 26.2%) [44] and TSP (9/79, 12% in its pilot test [41] and 23/251, 9.2% in its RCT [36]). Compared with the QTH RCT, participants in this study included fewer daily users, reported lower frequency of nicotine use, had lower levels of nicotine addiction as measured by time to first use upon waking, and showed higher motivation to quit. As a result, peer mentoring alone may not fully explain the observed differences in abstinence. While self-reported data from unblinded participants may not accurately reflect participants’ actual quit status, these findings suggest that peer mentoring has the potential to improve abstinence from e-cigarette use and warrant further research to assess efficacy. Additionally, focus groups provided valuable insights for future peer-mentored intervention design, such as increased use of videos to enhance interaction, opportunities for real-time support, and ways to extend the intervention duration to help sustain abstinence.

### Limitations

This study has several limitations. First, intervention participants were recruited from California only, and most had a strong desire to quit vaping. As a result, the findings of this pilot trial may not be fully generalizable to e-cigarette users in other regions or to those less motivated to quit. Second, abstinence data were self-reported and not biochemically validated. Third, the small number of pilot groups and focus group participants may not reflect the full range of participant experiences. The limited number of focus group participants also precluded the achievement of thematic saturation. Fourth, as a pilot study, the limited sample size does not allow the establishment of relationships between peer mentoring, engagement, and outcome variables. Future studies, such as RCTs or those using a multiphase optimization strategy design, are recommended to rigorously evaluate the efficacy of peer mentoring in supporting young people’s e-cigarette cessation.

### Conclusions

This study found that integrating peer mentoring into vaping cessation interventions delivered in groups on a social media platform is both feasible and acceptable. Participants rated both peer mentoring and the overall peer-mentored program highly. Self-reported 7-day point prevalence abstinence at the end of the intervention was very promising. These findings support a future full-scale trial to evaluate the efficacy of peer-mentored social media–based vaping cessation interventions.

## Abbreviations

QTH: Quit the Hit
RCT: randomized controlled trial
TSP: Tobacco Status Project
